# Supplementary material for: Causal relationships between anthropometric traits, bone mineral density, osteoarthritis and spinal stenosis: A Mendelian randomisation investigation
Source: Osteoarthritis Cartilage. 2024 Jun;32(6):719–29. doi: 10.1016/j.joca.2023.12.003 (PMC11954849; doi:10.1016/j.joca.2023.12.003)
Supplement: Supplementary file 1 — Supplementary material [file mmc1.docx]

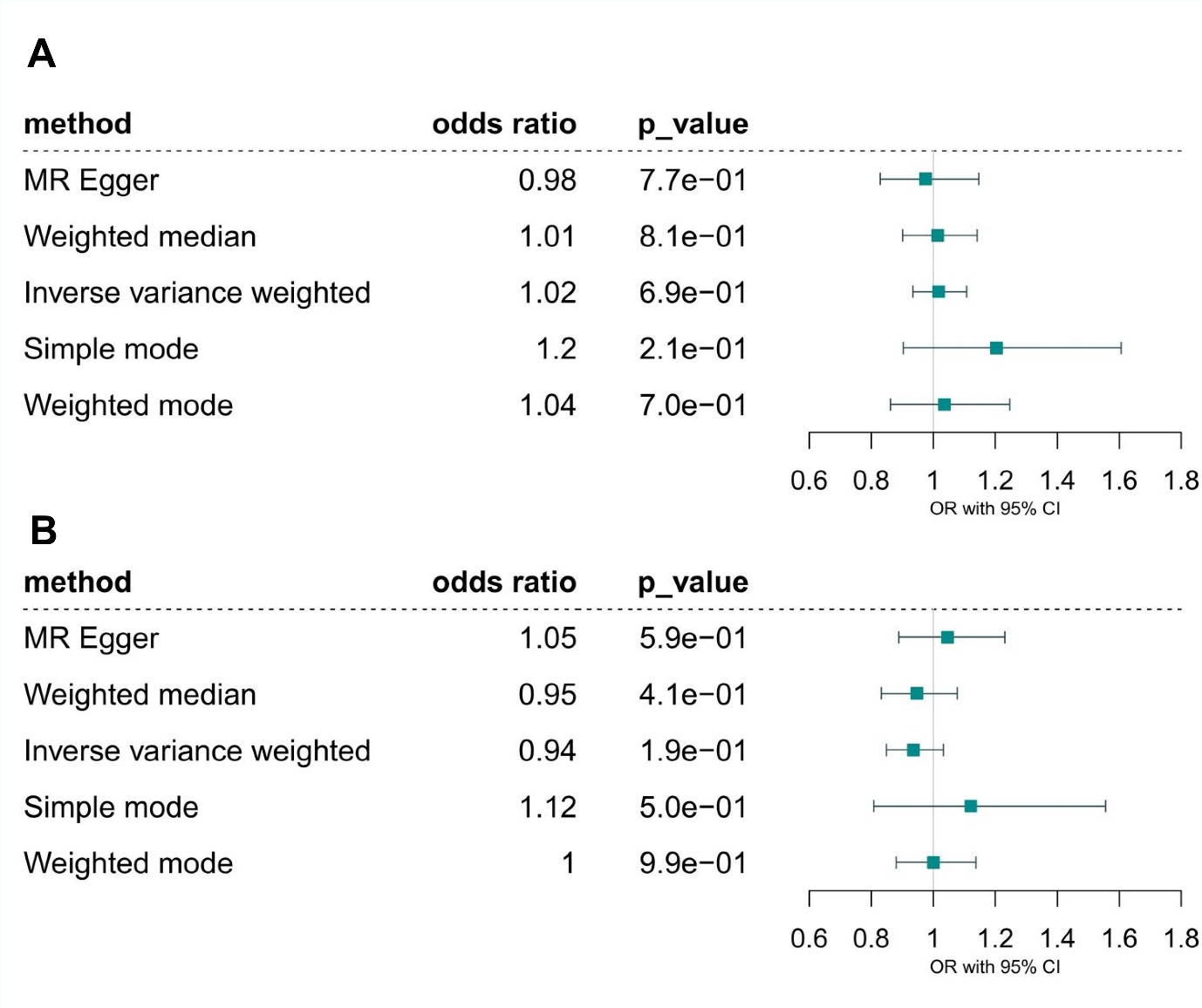
**Supplementary Figure 1.** Two sample Mendelian randomization results for the effect of genetic susceptibility for circulating calcium (**A**) and phosphate (**B**) on spinal stenosis (FinnGen). The odds ratios are scaled per SD increase in exposure.

**Supplementary Figure 2.** Scatter plot for the effect of genetic susceptibility for adiposity traits (**A** – BMI, **B** – hip circumference, **C** – waist circumference, **D** – waist-to-hip ratio) on spinal stenosis (FinnGen).


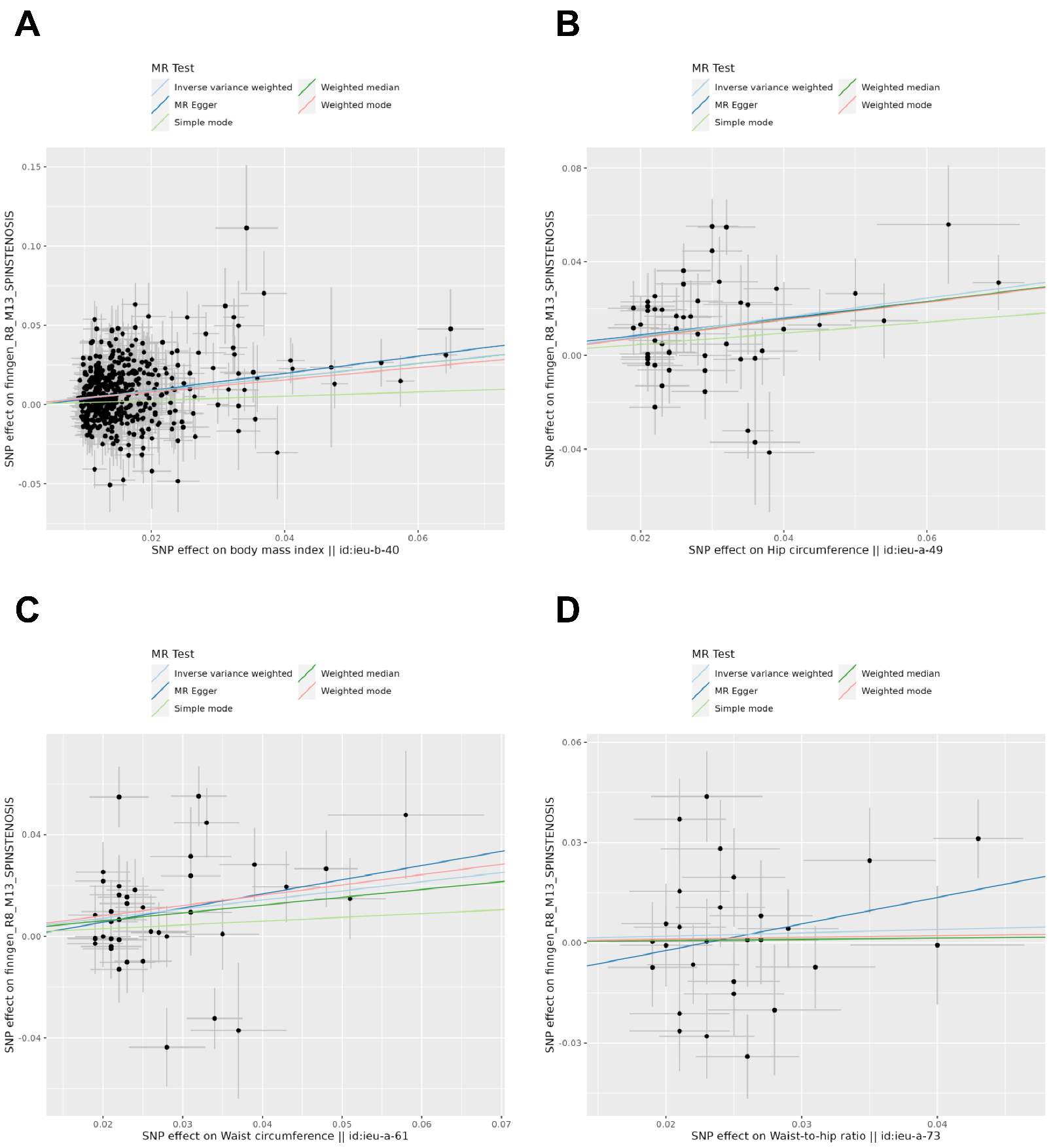


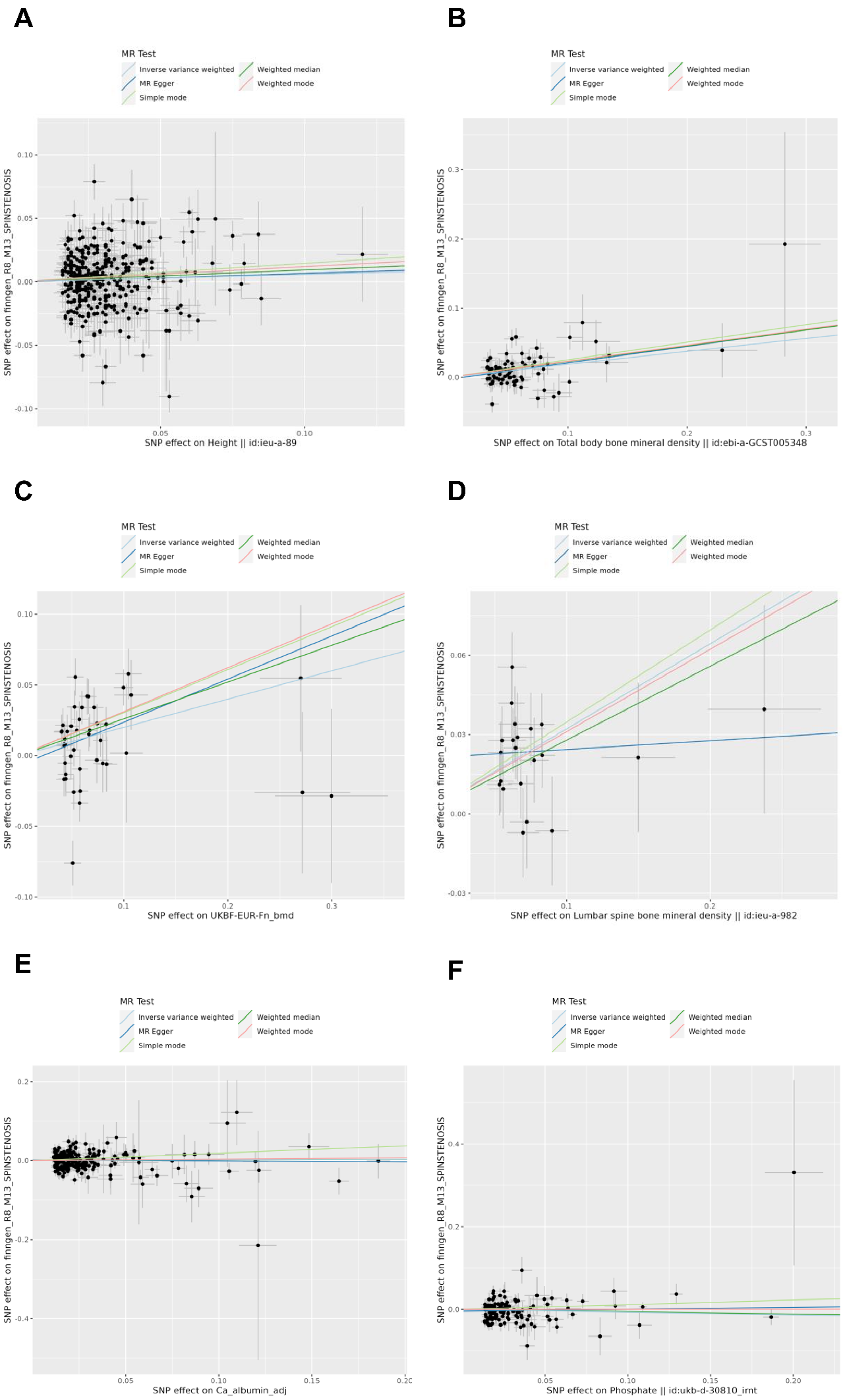
**Supplementary Figure 3**. Scatter plot for the effect of genetic susceptibility for skeletal traits (**A** – height, **B** – total body BMD, **C** – femoral neck BMD, **D** – lumbar spine BMD, **E** - albumin-adjusted circulating calcium, **F** – circulating phosphate) on spinal stenosis (FinnGen).

**Supplementary Figure 4.** Two sample Mendelian randomization results for the effect of genetic susceptibility for adiposity traits (**A** – BMI, **B** – hip circumference, **C** – waist circumference) on site-specific and all osteoarthritis. The odds ratios are scaled per SD increase in exposure.


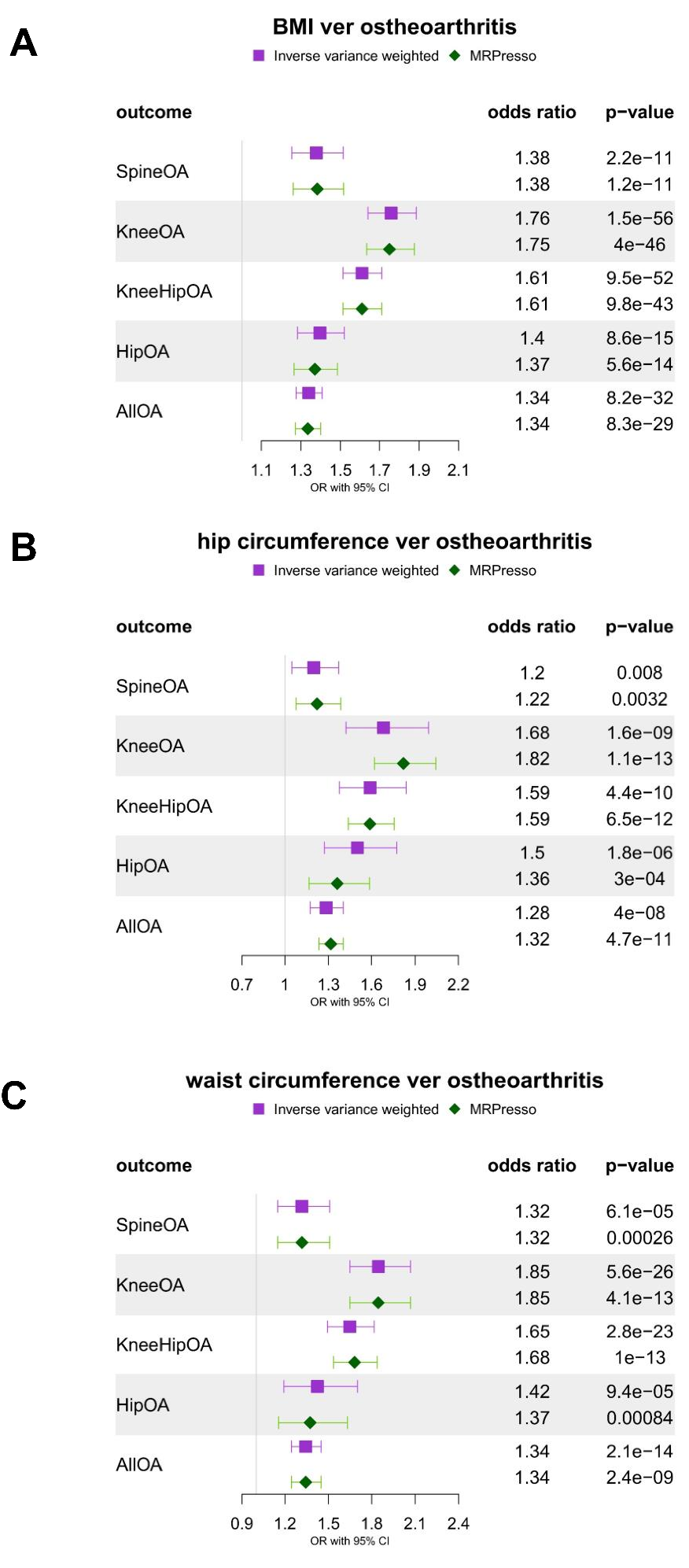


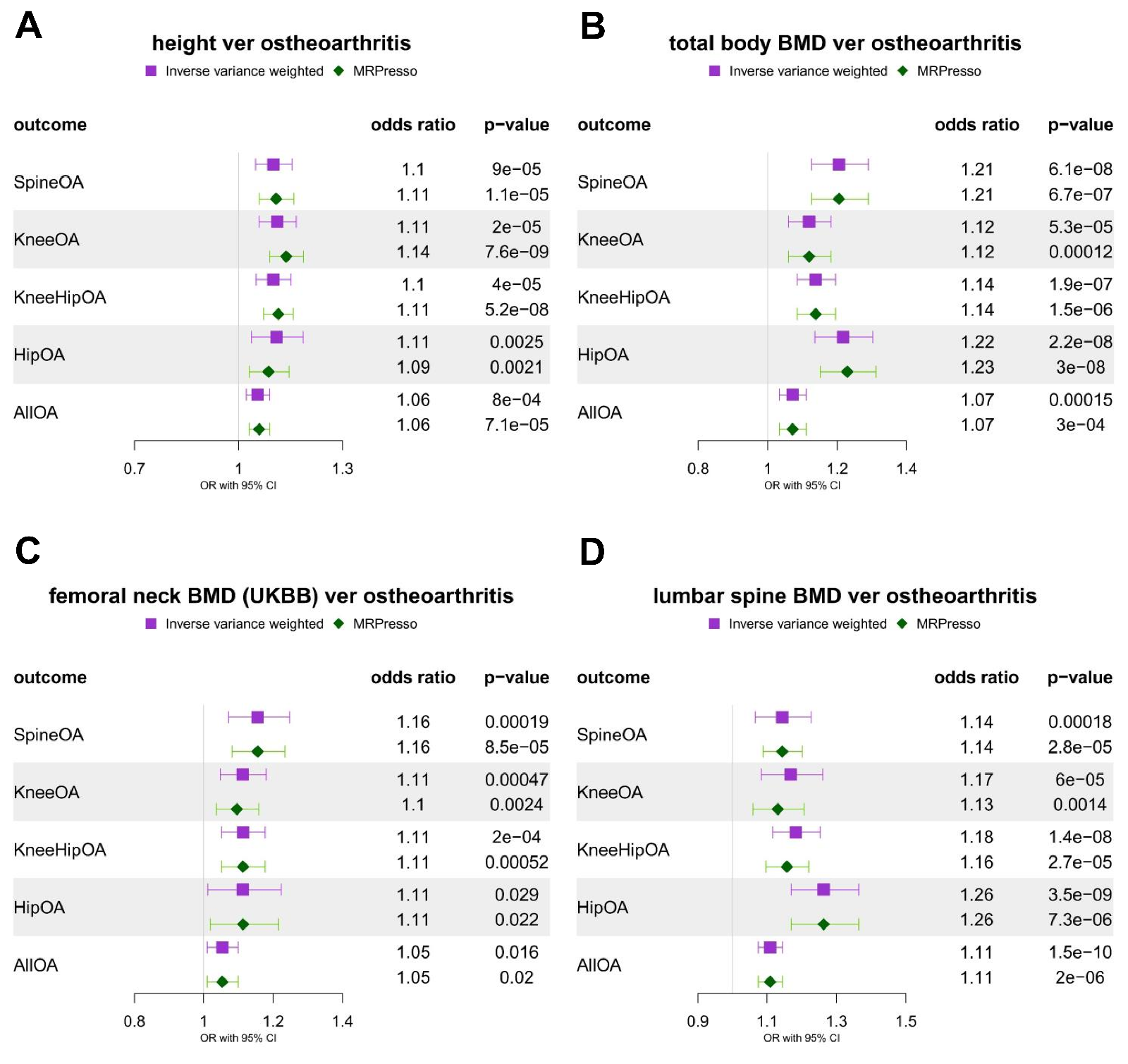
**Supplementary Figure 5.** Two sample Mendelian randomization results for the effect of genetic susceptibility for skeletal traits (**A** – height, **B** – total body BMD, **C** – femoral neck BMD, **D** – lumbar spine BMD) on site-specific and all osteoarthritis. The odds ratios are scaled per SD increase in exposure.

**Supplementary Figure 6.** Scatter plot for the effect of genetic susceptibility for osteoarthritis (**A** – all, **B** – hip, **C**- knee and/or hip, **D** – knee) on spinal stenosis (FinnGen).


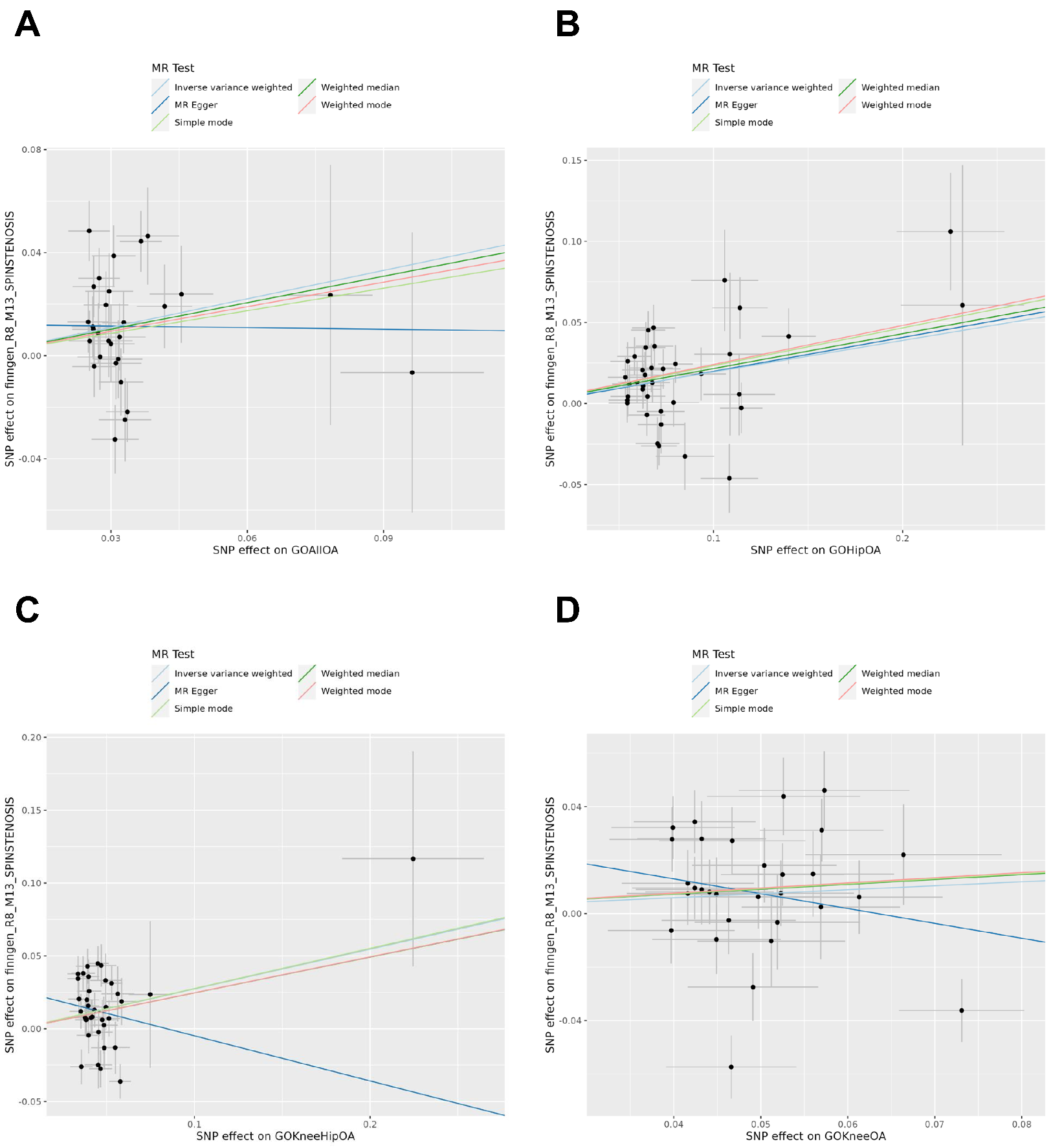


**Supplementary Figure 7.** Two sample Mendelian randomization results for the effect of genetic susceptibility for osteoarthritis on spinal stenosis (FinnGen). Plot compares results obtained using IVW/Wald ratio and outlier-robust MR-PRESSO method. The odds ratios displayed are scaled per doubling in odds of exposure.

**
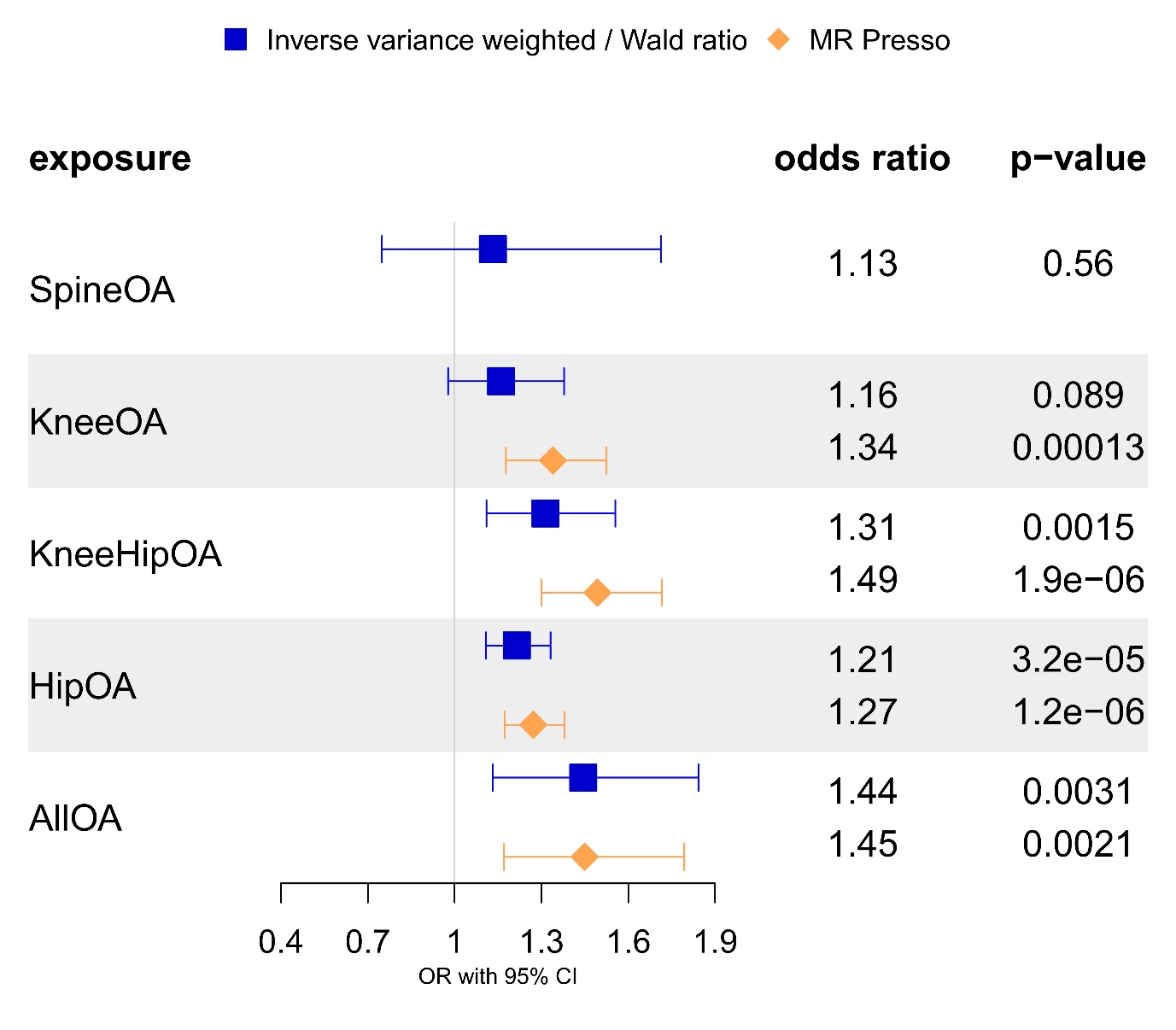
**


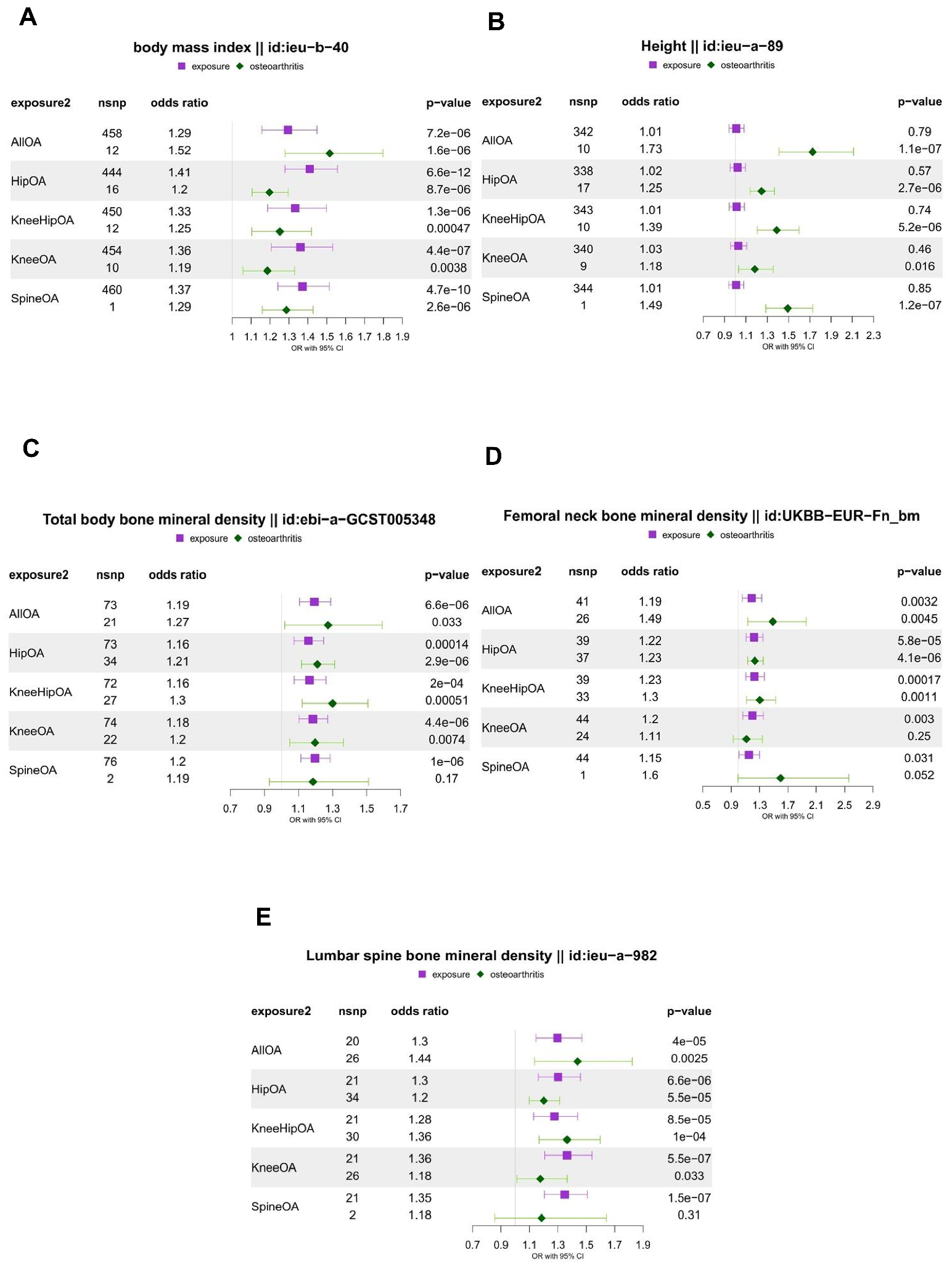
**Supplementary Figure 8.** Multivariable Mendelian randomization results for the jointly modelled effect of genetic susceptibility for: **A** - body mass index, **B** - height, **C** - total body BMD, **D** - femoral neck BMD, **E** - lumbar spine BMD and liability for osteoarthritis (all or site-specific) on spinal stenosis (FinnGen). The odds ratios are scaled per SD increase of BMI and doubling in the risk of osteoarthritis.

**Supplementary Figure 9.** Multivariable Mendelian randomization results for the jointly modelled effect of genetic susceptibility for risk factors (waist and hip circumference) and body mass index on spinal stenosis (FinnGen). The odds ratios are scaled per SD increase of risk factors.

**
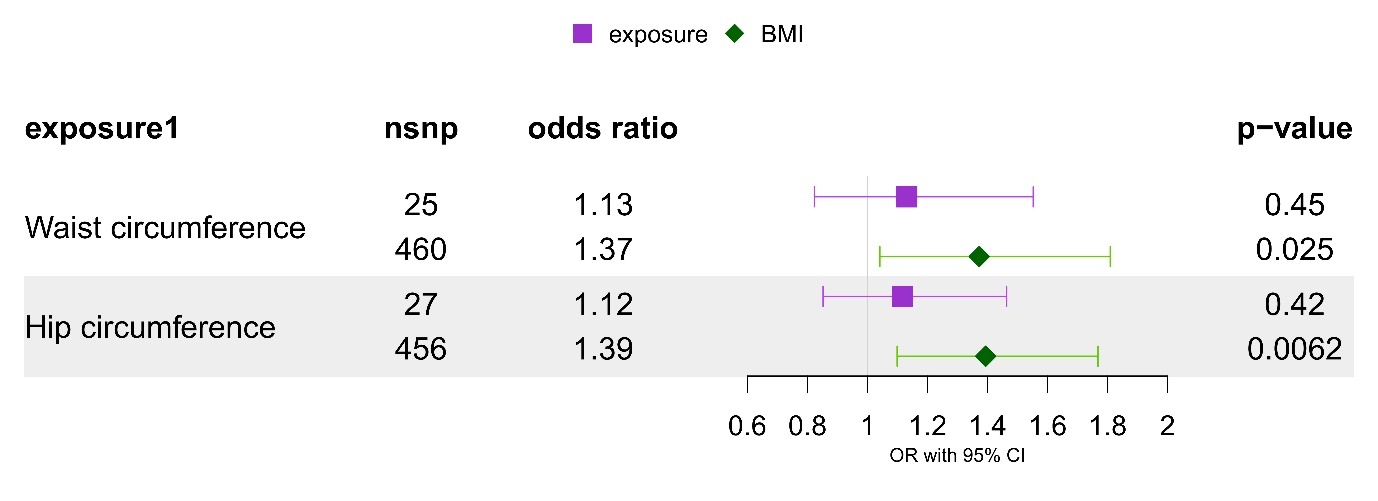
**

**
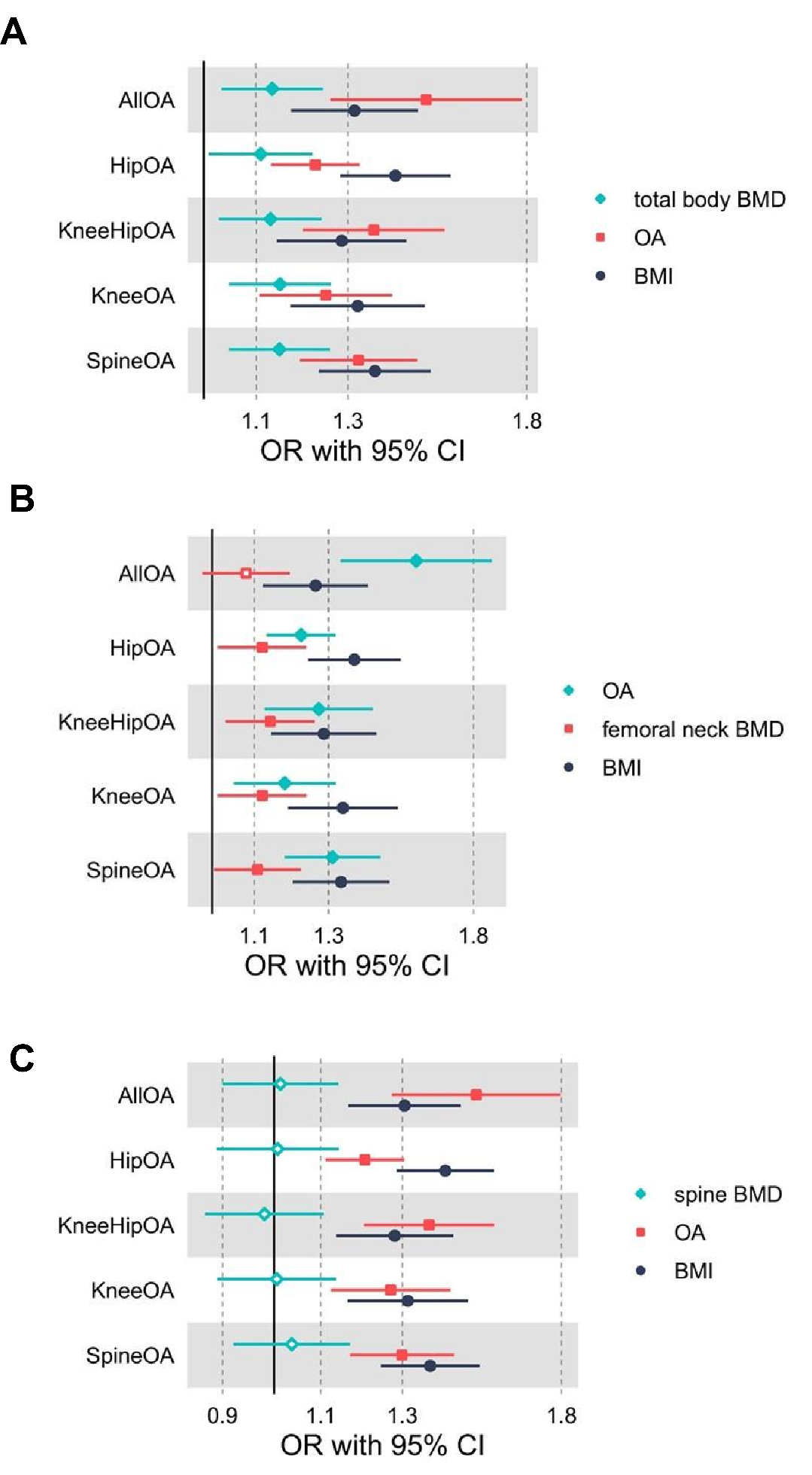
Supplementary Figure 10**. Multivariable Mendelian randomization results for the jointly modelled effect of genetic susceptibility for BMD (**A** – total, **B** – femoral neck, **C** – lumbar spine), BMI and liability for osteoarthritis (all or site-specific) on spinal stenosis (FinnGen). The odds ratios are scaled per SD increase of risk factors and doubling in the odds of osteoarthritis. Filled point shapes represent p-value < 0.05.
